# Advancing green recovery: Integrating one health in sustainable wildlife management in the Asia-Pacific Indigenous People and Local Communities

**DOI:** 10.1016/j.onehlt.2025.100969

**Published:** 2025-01-09

**Authors:** Nareerat Sangkachai, Anuwat Wiratsudakul, Delia G. Randolph, Maxine Whittaker, Acty George, Martin R. Nielsen, Nicholas Hogarth, Dirk U. Pfeiffer, Carsten Smith-Hall, P.O. Nameer, Latiffah Hassan, Gautam Talukdar, Tien Ming Lee, Vinod B. Mathur, Innocent B. Rwego, James Compton, Manon Mispiratceguy, Jianbin Shi, Amanda E. Fine, Illias Animon, Kristina Rodina de Carvalho, Andrew Taber, Scott Newman, Metawee Thongdee, Ladawan Sariya, Siriporn Tangsudjai, Waruja Korkijthamkul, Walasinee Sakcamduang, Sarin Suwanpakdee

**Affiliations:** a Faculty of Veterinary Science, Mahidol University, Thailand; b University of Greenwich, UK; c James Cook University, Australia; d Food and Agriculture Organization of the United Nations (FAO); e Department of Food and Resource Economics, Faculty of Science, University of Copenhagen, Denmark; f CIFOR-ICRAF, Nairobi, Kenya, University of Helsinki, Finland; g Centre for Applied One Health Research and Policy Advice, Jockey Club College of Veterinary Medicine and Life Sciences, City University of Hong Kong, Kowloon, Hong Kong, China; h College of Climate Change and Environmental Science, Vellanikkara, Kerala Agricultural University, India; i Department of Public Health, University of Missouri, Columbia, USA; j Wildlife Institute of India, Dehradun, India; k School of Life Sciences and School of Ecology, State Key Lab of Biological Control, Sun Yat-sen University, China; l National Biodiversity Authority of India, India; m College of Veterinary Medicine, Animal Resources and Biosecurity, Makerere University, Kampala, Uganda.; n TRAFFIC Global Office, Cambridge, UK; o School of Environment, Beijing Normal University, China; p Wildlife Conservation Society (WCS), USA

**Keywords:** Indigenous Peoples and Local Communities, Wildlife management, One Health, Zoonotic diseases, Food security, Poverty, Green recovery

## Abstract

Wildlife (in this paper: wild animals) deliver a crucial range of ecosystem services on human health and livelihood, particularly in Indigenous People and Local Communities (IPLCs). ‘One Health’ extends beyond just health; it also includes a comprehensive framework that can address wildlife and biodiversity conservation to enhance the well-being of humans, animals, and the environment with multisectoral collaboration. Therefore, integrating One Health principles into wildlife management was suggested in this review to improve the quality of life by reducing poverty, improving food security, and preventing zoonotic diseases in IPLCs. The relationship between wildlife interactions and the emergence of pathogens that can be transmitted between wild animals, domestic and production animals, and humans underscores the need to incorporate a One Health approach to mitigate risk. This integration will also contribute to conserving wild animals and their habitats and biodiversity for ecosystem balance. This review highlights the importance of One Health in supporting sustainable wildlife management to achieve a green recovery through policies and actions based on global and national regulatory frameworks, development of local policies with community engagement, risk assessment and communication, sustainable wildlife use practices, and conducting research and innovation. Monitoring and analyzing data on supply chains and economic values can serve as a decision-support tool for sustainability wildlife management. A theory of change for sustainable wildlife management and enhancing human well-being is proposed using the One Health approach. All these activities must respect local cultures and traditions, ensuring that One Health and community-based approaches effectively benefit local communities.

## 1 Introduction

In this study, wildlife is defined as living animals that are neither human nor domesticated. However, we have also included wildlife caught or bred for farming as a definition of captive wildlife. They play a crucial role in providing ecosystem services [1], including 1) provisioning services for food, income, and medicine, 2) regulating services such as disease spillover between animals, including human species, and 3) cultural services relating to cultural and spiritual experiences [2]. Wildlife species are used as a food source for humans, contributing to the food security of millions of people globally, particularly in low- and middle-income countries [3]. Wildlife species also contribute to global food systems' long-term sustainability and health by preserving genetic diversity [4].

The connection between wildlife use and the emergence of zoonotic diseases underscores the crucial importance of sustainable wildlife management in preventing the transmission of diseases to humans and livestock. Most emerging infectious diseases (EIDs) in humans originate in animals, particularly wildlife [5]. Additionally, remote rural areas, particularly in low-income and middle-income countries, are routinely exposed to zoonotic pathogens because of close contact with livestock and wildlife. This suggests a need to improve disease surveillance and integrate a systemic approach to health based on the understanding that humans, animals, and ecosystem health in remote rural areas are intertwined [6].

The One Health approach explicitly recognizes the interconnection between the health of people, animals, and shared environment and advocates a collaborative, multispectral, and trans-disciplinary approach working at the local, national, regional, and global levels to achieve optimal health outcomes [7]. This approach collaboratively enhances well-being and addresses challenges related to health and ecosystems. It seeks to fulfill the collective demands for nutritious food, clean water, energy, and air while taking decisive action on climate change and advancing sustainable development efforts [8]. One Health also now emphasizes ecological value and wildlife conservation, bringing a holistic socio-ecological perspective for resolution [9]. This emphasizes the need for an interdisciplinary and intersectoral approach to ecosystem restoration, improving human well-being, and mitigating disease risk. Such efforts would also contribute to the sustainable management of wildlife and diverse natural resources, helping to achieve several Sustainable Development Goals (SDGs): 1) SDG 1 - no poverty by reducing poverty, 2) SDG 2 - zero hunger by supporting food security and nutrition, 3) SDG 3 - good health by providing for good health and well-being, and 4) SDG 15 - life on land by conserving terrestrial habitats and biodiversity [10]. This is especially important for Indigenous Peoples and Local Communities (IPLCs). The Asia-Pacific region's biodiversity and ecosystem services are essential for human well-being and sustainable development. However, they are increasingly at risk of ecosystem degradation from human activities, climate change, and habitat loss. For example, about 80 % of the region's natural ecosystems have been degraded, directly impacting both wildlife and IPLCs [11]. IPLCs have profound connections to their territories and extensive knowledge of seasonal and ecological cycles [12]. In addition, IPLCs maintain deep relationships with diverse ecosystems such as grasslands, marine environments, and freshwater systems. Each of these ecosystems provides unique resources and challenges for IPLC livelihoods and requires tailored approaches for sustainable management.

The concept of green recovery is a strategic and policy-oriented approach to rebuilding the economy and society while prioritizing environmental sustainability [13]. This approach is aimed at addressing the economic challenges brought not only by the COVID-19 pandemic but also emphasizes the need for socio-economic growth that improves IPLCs livelihoods while protecting the environment and maintaining the quality of natural resources [14]. Green recovery also promotes sustainable wildlife management and tackles the environmental factors contributing to zoonotic disease spillovers. A holistic solution is essential to achieve these goals [9].

**Fig. 1:**
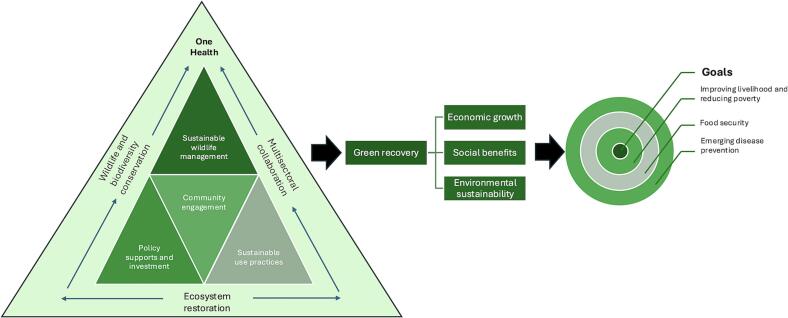
A Framework illustrating the role of One Health in promoting sustainable wildlife management toward achieving green recovery. (For interpretation of the references to colour in this figure legend, the reader is referred to the web version of this article.)

This narrative review identified knowledge and action gaps in the relationship between sustainable wildlife management, holistic resolution, and the One Health approach for improving human health and the livelihoods of IPLCs as green recovery. The aim is to advocate policy support and investment, community engagement [15,16], and sustainable wildlife use practices [17] that are a part of ecosystem restoration strategies [15] and support sustainable wildlife management. Those activities will enhance multisectoral collaboration, align with promoting wildlife and biodiversity conservation as the holistic One Health concept [9], and support green recovery and goals for improving the quality of life of the IPLCs by improving their livelihood, food security and preventing zoonotic disease transmission at humans-domestic animals-wildlife interface (Fig. 1).

## 2 Indigenous Peoples and Local Communities in the Asia-Pacific region

Indigenous Peoples are those whose ancestors have inhabited an area prior to the establishment of modern state borders. They have distinct languages, cultures, and social and political institutions that may vary considerably from mainstream society [18]. Local Communities are the broad term that includes people who reside in relatively remote geographical areas with similar requirements and objectives when making decisions that affect their lives [19] or people who identify as a collective group who contribute to defining territory and culture through time [20]. The Asia-Pacific region is home to more than 260 million Indigenous Peoples, or 70 % of the total Indigenous Population in the world [21]. Of all Indigenous Peoples in the region, 72.8 % live in rural areas [22]. IPLCs constitute the majority of the population in certain regions (Supplementary Table S1). A wide variety of IPLCs, ranging from coastal fishing communities to highland agricultural societies. Each group has developed distinct systems of managing local wildlife and natural resources, often shaped by their specific ecological and cultural contexts (Supplementary Table S2). They serve as the guardians of ancestral knowledge related to biodiversity and play significant roles in protecting biodiversity [23]. Through their practices and knowledge, IPLCs have significantly contributed to the sustainable use, conservation, and improvement of forests for food, biodiversity, health, and livelihoods [21]. Nevertheless, they may also have mistaken beliefs about wildlife and sometimes use wildlife unsustainably [24]. Despite the importance of Indigenous knowledge for sustainably managing forests and biodiversity, Indigenous Peoples are among the most marginalized groups in the Asia-Pacific region [25].

## 3 Wildlife in the livelihoods of Indigenous Peoples and Local Communities

**Fig. 2:**
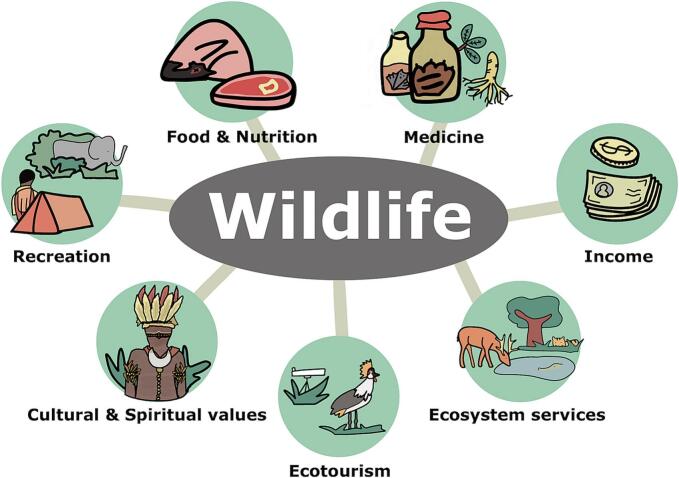
The contributions of wildlife to Indigenous Peoples and Local Communities in the Asia-Pacific region.

Wildlife is an important resource with significant nutritional, economic, medicinal, cultural, and recreational values (Fig. 2). Wildlife consumption has been a part of Asia-Pacific culture for over 40,000 years, with each community having its own beliefs and practices [26]. Hunting and trading could have both economic and cultural drivers, which can contribute to unsustainable harvesting [27]. In remote forested areas with high levels of malnutrition, wild meat supports household dietary needs and provides crucial nutrition and subsistence income [28,29]. However, there are significant research and knowledge gaps, including scarce hunting data at the site level and a lack of regional comparative analyses. This makes it difficult to estimate the extent of IPLCs' dependence on wild meat and the potential impact on their well-being if access to wild meat declines without suitable alternatives [30]. Appropriate policymaking becomes significantly challenging when designing effective strategies that address the needs and vulnerabilities of IPLCs.

Traditional hunting has been an important part of Indigenous People's lives for centuries, with hunting motives varying by location [31]. Wild meat serves as a significant income source for rural households, and hunting in Asia-Pacific communities is often opportunistic and indiscriminate [32]. Wildlife products go through complex value chains from the forest to the consumer [33], and economic and socio-cultural factors impact the demand and supply of wildlife trade. Some wildlife products are considered luxury goods, such as rhino horns or ivory-based art [34]. Research in Vietnam found significant differences in awareness of endangered wildlife among IPLCs. Knowledge of the law influenced actions, with gender being a major factor in awareness levels. Many Indigenous people showed a positive attitude toward protecting endangered species affected by their community and culture [35].

However, Southeast Asian biodiversity is highly vulnerable to habitat loss. Protecting the region's forests and biodiversity demands integrating social issues, like employment in local communities, into conservation planning [36]. Additionally, human-wildlife conflict (HWC) affects the livelihoods of local communities. Residents near community forests in Nepal have reported that wild predators are killing their livestock, including chickens, sheep, and goats, resulting in significant financial losses [37].

## 4 Indigenous Peoples and Local Communities involved in wildlife management

IPLCs can play a crucial role in sustainable wildlife management due to their traditional knowledge, cultural values, and close relationship with nature [38]. Community-based wildlife management models have shown significant economic contributions to local economies, providing communities with direct financial benefits, employment opportunities, and improved infrastructure [39]. However, their impacts and economic benefits should be evaluated, and the evaluation method should be used in the same way for comparison in similar models [40].

In the Asia-Pacific region, legal and regulated commercial wildlife farming is extensively promoted to decrease the pressure from hunting and illegal sourcing of wildlife and wildlife products, support conservation, and promote sustainable wildlife use [43]. Wildlife farming often supports food nutrition and household income. For example, Vietnam has developed commercial wildlife farming, such as civets, porcupines, and snakes [41]. In some cases, they also promote economic development for companies and state-owned businesses, such as the deer wildlife industry in China [42]. The industry supports numerous state-owned enterprises and private companies engaged in producing wildlife products like meat, leather, and traditional medicines. The economic benefits extend to local communities through job creation and business opportunities, contributing to regional economic development while attempting to reduce pressures on wild populations [43].

However, the performance and sustainability of wildlife farming have been questioned due to changes in customer preferences, economic conditions, and sociocultural factors [44]. Challenges include changes in consumer popularity, higher prices [45], and limitations in success in husbandry and breeding. For these reasons, the offtake of wildlife from the forests continues to persist for replacement/replenishment of breeding stocks and laundering from illegal sources [46].

## 5 Risks and drivers of the zoonotic pathogen transfer at the human-wildlife interface

A study of fifty-four wild animal species by the Convention on Migratory Species found that they carry at least one of sixty pathogens transmissible to humans, posing a disease risk. The type of animal greatly influences the risk of zoonotic diseases [47]. Understanding pathogen spillover drivers and their impacts is crucial. These include pathogen and wildlife characteristics, environmental changes, and wildlife consumption and trade [48]. In contrast, zoonotic diseases in humans and domestic animals can also transmit the pathogen back to wildlife populations. For example, SARS-CoV-2 may spread from humans to white-tailed deer through feeding, exposure to contaminated secretions, and fomites [49]. Healthy ecosystems are crucial for the planet, helping to prevent disease spillover and combat climate change. They also support food production, purify air and water, recycle nutrients, and provide genetic resources and habitats [50].

### 5.1 Land-use changes

Human-induced land-use changes are the primary drivers of various infectious disease outbreaks and emergence events [51]. Agricultural expansion in forested areas leads to the shrinking and fragmentation of wildlife habitat, facilitating pathogen spillover [52].

The linkage between zoonotic outbreaks and declines in biodiversity and ecosystem integrity has been observed in the Asia-Pacific region [53]. However, most studies are based on modeling or meta-analyses rather than actual field data [54]. This is partly due to difficulties in large-scale and long-term data collection [55]. The relationship between degraded environments and increased risk is not well understood, and identifying the living conditions of a disease-causing microorganism in its environment remains a challenge [56].

### 5.2 Subsistence hunting

Due to several factors, subsistence hunting can increase the risk of zoonotic disease spillover. Activities like subsistence hunting and handling wildlife can expose people to pathogens. Regular exposure to zoonotic pathogens may confer immunity, as shown by a 2.7 % seroprevalence of SARS Coronavirus antibodies in residents near bat caves in China. Traditional hunting practices and the consumption of bushmeat are deeply embedded in local cultures, hindering public health interventions aimed at reducing zoonotic risks [57].

### 5.3 Wildlife farming, wildlife markets, and international trade

Commercial farming of wildlife species may increase spillover risks, especially if the farmed species are hosts for zoonotic pathogens. Increased contact with humans along wildlife trade chains is linked to higher infection risks [58]. Unregulated wildlife farming should be concerned with the risks of zoonotic diseases spilling over. In particular, failing to implement biosecurity protocols like isolating wild-caught animals before their introduction to farms can enhance the spread of pathogens between wildlife and humans [58]. Commercial civet farming in Vietnam does not provide adequate evidence concerning quarantining newly acquired wild-caught civets intended for restocking farms [59]. In China, there are legitimate concerns regarding animal traceability, quarantine protocols, and the supervision of these wild-caught animals by veterinarians at the farms. This issue is reminiscent of the situation in China following the COVID-19 pandemic. Implementing these measures is essential to safeguarding the health and welfare of wildlife [60]. Conditions such as high stock volume, unsanitary environments, and overcrowding in wildlife farms elevate spillover risks. Poor living conditions of workers may increase their vulnerability to zoonotic disease transmission due to poor nutrition and compromised immune systems [61]. Additionally, personal protective equipment (PPE) and knowledge of biosecurity are needed for farm workers [62].

The transportation of hunted wild species to markets and their close contact with humans and livestock can increase the risk of pathogen transmission [29]. Poorly regulated wildlife markets with high animal densities and inadequate sanitation amplify infection risks. Additionally, the unregulated international wildlife trade poses a significant exposure risk to zoonotic pathogens [63].

## 6 Green recovery in sustainable wildlife management

Green recovery is intrinsically connected to ecosystem restoration, such as wildlife habitat restoration, biodiversity conservation, community engagement, and sustainable land use [14]. Those have significantly enhanced local communities' economic opportunities, job creation, and IPLCs' livelihoods through ecotourism, sustainable wildlife harvesting, agricultural production, fisheries management, and others [64]. Notably, A case study promoting wildlife conservation in Shennongjia National Nature Reserve (NNR) in China highlights the significant growth in ecotourism revenue, which increased from 1,585,000 USD in 2005 to 3,390,000 USD in 2010. Some of these earnings benefited the local community [65]. Furthermore, following the emergence of SARS-CoV-2, the mitigation measures for zoonosis focused on wildlife resources and trading. The COVID-19 pandemic and its severe consequences impacted people's livelihoods, health, and food systems, with over a billion global workforce at risk of losing their livelihoods [66]. During lockdowns, some people could not earn an income for their families, which affected their food security [67]. The opportunities for rural households to work as hired labor also significantly decreased. Therefore, to sustain wildlife management to support green recovery for IPLCs, our recommendations will focus on top-to-down and bottom-up policies, community-based practices, and sustainable use practices (Fig. 1) to promote green recovery, as follows:

### 6.1 Policy supports and investment: Global and national frameworks

The Kunming-Montreal Global Biodiversity Framework (KMGBF), established during the 15th Conference of the Parties (COP15) of the Convention on Biological Diversity (CBD), aims to reverse biodiversity loss and protect ecosystems, which indirectly contributes to reducing the risk of zoonotic diseases [68]. Additionally, the Convention on International Trade in Endangered Species (CITES) monitors the trafficking of animals and plants to prevent overexploitation and minimize the transmission of diseases from animals to humans [69]. In contrast, the Convention on Migratory Species (CMS) concentrates on safeguarding the habitats of migrating species, playing a vital role in sustaining balanced ecosystems that protect against the rise of zoonotic diseases [70]. Therefore, the frameworks established at the national level should align with those global frameworks and support the goals of green recovery.

Restoration investment can transform degraded landscapes into good environmental health, generating job creation and fostering business growth. The national governments should develop funding strategies that shift from harmful land uses to sustainable landscapes. Therefore, to gain political support, a successful restoration plan should focus on three key areas: environmental goals, economic benefits, and social outcomes [16]. Partnerships with NGOs and international organizations can enhance the avenues for effective implementation and funding support [71].

Additionally, policies that enhances capacity building and collaboration/coordination on disease surveillance at the human-domestic animal-wildlife interface will provide an effective early warning system for disease prevention [72]. Table 1 outlines examples of objectives and key barriers of policies and measures to support local communities for zoonotic disease prevention.

**Table 1:** Policies and Measures with Objectives and Key Barriers for Zoonotic Disease Prevention.

	Disease prevention	
**Objectives**	Key barriers and suggestions	References
Understand the ecosystem degradation impact on disease spillover	Land use changes in tropical forest regions are one of the key risk factors spatially associated with disease spillovers from wildlife into humans.	[73,74]
	A need to understand the role of ecosystems in the regulation of diseases, especially in regions and countries with a higher risk for/role in zoonotic disease outbreaks.	[75,76]
Reduce urban demand for wild meat; Reinforce controls on wildlife trade	Banning wildlife trade may help prevent the emergence of zoonosis, but some scientists have indicated that this affects food security, conservation, economics, and public health.	[[77], [78], [79]]
	Regional wildlife poaching and trafficking due to porous borders and weak enforcement of regulations.	[[80], [81], [82], [83]]
	Observing the demand reduction side to see how to change consumer behavior in order to change the demand for wild meat.	[83,84]
Develop sustainable and safe local food systems; Support risk assessment	Any restrictions need to be based on a sound understanding of the value chains through which wild animals and their products are traded and processed. Risk assessments are needed to quantify risk of pathogen emergence and based on an understanding of the underlying risk pathways.	[29,85]
	The tools and approaches needed to identify, assess, and manage the risks that could enable the sustainable provision of the benefits of sustainable wildlife management to Indigenous Peoples and local communities	[86,87]
	A survey is being conducted to identify national disease surveillance activities in wildlife and to understand the data generated by both government and non-government entities for risk mitigation measures and building a more holistic One Health approach.	[88,89]
	Risk assessments need to have clear public health data/ epidemiological data and taxa-specific data to be effective, particularly for wildlife.	[29,63,90]

### 6.2 Community engagement

#### 6.2.1 Local policies, customary laws, and spiritual and cultural values

Locally applied policies and regulations should allow IPLCs to increase wildlife benefits and income sustainably. Legal and institutional frameworks can empower IPLCs to sustainably manage wildlife and natural resources. Studying Indigenous communities in Thailand and Russia provides valuable insights for developing effective policies incorporating Indigenous perspectives. It emphasizes the importance of self-determination, cultural preservation, and land rights, highlighting the need for legal frameworks that prioritize Indigenous involvement in climate and sustainability efforts [91]. Decentralization was suggested so that IPLCs could be involved in the issue of local policies [92]. Addressing the root causes of overharvesting is crucial for IPLCs. Such policy approaches should also consider the market chain, harvest, and wildlife conservation [93]. The commercialization of the wild meat trade can be successful through multiple actions, including education, regulations, and law enforcement [30].

Customary law can help with sustainable management but doesn't always align with national legislation. Local understanding can improve policy implementation. Local regulations can be carefully designed to consider their impact on the livelihoods of IPLCs. Biosecurity is essential for managing wildlife trade to reduce the spread of infectious diseases [94]. Beyond the need for appropriate policies and regulations is the need to ensure they are applied once they are put into effect. A lack of implementation capacity and local support has been known to render laws effective only on paper [95]. To implement local policies and regulations more effectively, understanding and gaining the buy-in of local communities is important [96].

Understanding and respecting cultural values and knowledge is crucial when working with Indigenous Peoples on wildlife use and supporting sustainable wildlife management in their Local Communities [87]. Buddhist beliefs play a key role in local conservation. A “sacred” value orientation valued their spiritual beliefs highly and showed greater tolerance for wildlife, especially species with religious significance like Elephants and Tigers in Bhutan [97]. Research on cultural values can provide knowledge of the relationship between Indigenous Peoples and wildlife [98]. Such insights are crucial for policymakers to design regulations informed by and responsive to local socio-cultural norms, emphasizing the need for bottom-up participative processes [87].

#### 6.2.2 Risk assessment and communication

Good practices of wildlife use along the wildlife supply chain should promote safety from disease risk by wildlife exposure [99]. This can be achieved through risk assessments to measure the risk of pathogen emergence and develop enforceable risk mitigation measures based on understanding the underlying risk pathways [29]. Prevention of Emerging Infectious Diseases (EID) occurrence will reduce the chance of socioeconomic impact of the COVID-19 pandemic [100]. Risk communication is a crucial tool in approaching Indigenous Peoples and Local Communities (IPLCs) for disease prevention in the wildlife sector [101]. Risk communication and community engagement (RCCE) in the outbreak literature is a useful guideline for practitioners [102]. Establishing a comprehensive risk communication strategy is important when engaging communities that interact regularly with wildlife. Successful risk communication with Indigenous populations involves aligning messages with cultural beliefs, engaging the target audience in message design, using trustworthy spokespeople, effective communication materials and channels, and ensuring message clarity [103].

### 6.3 Sustainable wildlife use practices

#### 6.3.1 Promoting rational exploitation of wildlife

Wildlife farming can be an alternative solution to advocate for change in local communities. It provides a food source, increases the economic value of the supply chain, and can be managed in protected areas in some countries [97]. Conservation professionals and policymakers should support current practices to safeguard biodiversity while enhancing the well-being and livelihoods of local residents [104]. Caution should be exercised regarding the replacement of some wild-caught wildlife sold in wildlife farming, which has often been reported [59,105]. There is a need for enhanced law enforcement on wildlife carcass management, and experts can assist the government in ensuring that farmers comply with regulations. The imbalance between wildlife farming production and the high demand for wildlife consumption has led to increased hunting and harvesting of these animals [46]. Promoting agricultural land in local communities and using alternative protein sources may help reduce the overexploitation of wildlife [106].

Community-based ecotourism (CBET) is also an alternative solution that is a sustainable approach to developing areas by integrating community tourism, ecotourism, and environmental education. In CBET, the community participates in tourism-related economic activities, and most or all tourism businesses are owned and managed by the community. This involvement helps the community to conserve natural resources, generate income, and enhance the quality of life for local residents. This sets CBET apart from ecotourism, where community involvement in ecological areas is limited [107]. In Vietnam, CBET contributed to mutual benefits and income for IPLCs that suggested policy and management implications, such as raising stakeholder awareness, securing community participation, and establishing fair revenue-sharing mechanisms [108]. However, IPLCs may see CBET as a potential economic option. Still, they may encounter major challenges such as insufficiently trained staff, poor infrastructure, lack of financial support, limited tourism knowledge, inadequate government support, and weak cooperation among local stakeholders [109]. Thus, enhancing the local capacity is needed to support sustainable wildlife use practices.

Wildlife farming and ecotourism may not fully benefit local communities as intended. Instead, the profits may disproportionately flow to large enterprises or even criminal operations. This complex issue of inequity deserves careful consideration, particularly in developing countries [110].

#### 6.3.2 Raising awareness and education

The awareness program is a successful example of conservation that involves community participation in wildlife conservation activities in and around protected areas. In Hong Kong, a successful student-led outreach program has effectively increased knowledge about horseshoe crabs' biology and ecology while fostering positive attitudes toward conservation within communities when implementing those activities. The lessons learned from this program can serve as a guide for similar initiatives in other locations. However, this program used effort to input education for the long term and used multi-disciplinary learning components [111]. Furthermore, providing income-generating opportunities and alternative livelihood training related to wildlife conservation for local communities could help improve long-term wildlife conservation efforts [112].

#### 6.3.3 Research and innovations

It is crucial to evaluate the potential of innovations through technical, institutional, social, and existing technologies, including traditional knowledge, to transform current agri-food systems and improve the lives of IPLCs by restoring landscapes and strengthening the One Health approach on the ground. For example, Data collection and management made use of the Spatial Monitoring and Reporting Tool (SMART) as well as the Wildlife Health Intelligence Platform (WHIP). One Health approach, driven by local communities, was utilized to set up Wildlife Health Surveillance in Cambodia, Lao PDR, and Viet Nam as part of the WildHealthNet project [88]. Also, an integrated socio-ecological system for snow leopard conservation in Asia involves cross-border collaboration, peace parks, transnational conservation policies, improved livestock management, population control readiness, climate change mitigation, transboundary habitat conservation, environment-friendly trade corridors, and sustainable tourism [113]. The research will provide information to policymakers to develop appropriate policies, funding, and investment for sustainable wildlife management for IPLCs. The implementation of the Saiga Memorandum of Understanding (MOU) by governments, with support from the Convention on Migratory Species (CMS) and the Convention on International Trade in Endangered Species (CITES), received strong backing from dedicated non-governmental organizations (NGOs) and the scientific community involved in saiga research at both national and international levels. This collective effort resulted in a robust conservation network that successfully attracted funding for MOU implementation, as well as for international workshops and field projects [114].

#### 6.3.4 Data monitoring and analysis

Data monitoring on household incomes, local economy, food security, wildlife use, wildlife trade, etc., will provide vital information. Analyzing wildlife value chains helps estimate wildlife value and identify stakeholders at risk of zoonotic pathogen exposure [115]. Sharing study results and recommendations with policymakers and resource partners is crucial [116]. The long-term line transect distance sampling surveys in Cambodia have studied wildlife populations and can predict trends in growth or decline, particularly among threatened species [117,118]. Efforts to monitor illegal wildlife trade in Indonesia aim to achieve the sustainable development goals (SDGs) by 2030. The findings indicate a remarkably stable situation concerning the illegal wildlife trade, which highlights the ongoing challenges [118].

## 7 One Health and community-based approaches

“Health” is influenced by a combination of social and ecological factors that create threats, vulnerabilities, resources, and abilities. These factors determine how effectively an individual, population, or system can navigate their lived reality [119]. It is essential to address the fundamental knowledge gaps identified in this review to enhance the connections among health, wildlife management, and the well-being of IPLCs. Wildlife health is closely linked to conservation efforts, as demonstrated by initiatives such as wild bird surveillance programs. These programs provide early warnings to key sectors, including the poultry industry, agricultural authorities, wildlife agencies, conservation programs, wildlife rehabilitators, zoos, and public health officials, regarding potential outbreaks and the spread of highly pathogenic avian influenza (HPAI) viruses [119,120]. This interconnected approach highlights that maintaining the health of wildlife populations not only protects ecosystems but also plays a vital role in safeguarding both human and animal health on a larger scale [119,120]. The holistic One Health approach offers a unique opportunity to involve IPLCs in wildlife conservation by integrating traditional knowledge and promoting equitable resource distribution [121]. Collaborative efforts between ministries can facilitate information sharing, thereby addressing wildlife use, poverty reduction, food security, and cultural heritage [122]. Recognizing the rights of IPLCs and fostering their collaboration with policymakers and conservationists is crucial to ensure sustainable wildlife management that balances socio-economic needs and conservation goals [123,124].

Additionally, capacity building must be enhanced to cope with emerging diseases, particularly in supporting and establishing interdisciplinary collaboration work. A leadership training program may be useful for local policymakers [125]. Also, citizen science and integrating traditional knowledge with the latest scientific knowledge can be developed [126] to support the accessibility of predictive information on emerging infectious diseases of wildlife origin for future disease prevention [127]. Thus, a holistic resolution and One Health approach can enhance sustainable wildlife management and the well-being of local communities, ultimately benefiting human, animal, and environmental health, as illustrated in Fig. 1.

**Fig. 3:**
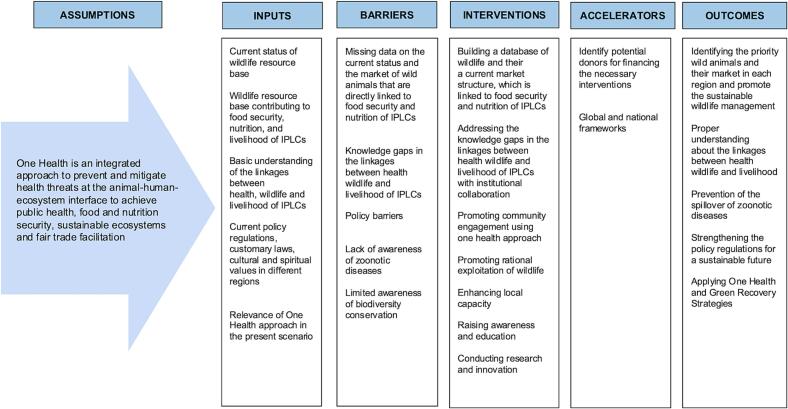
Theory of Change for sustainable wildlife management and human well-being.

To ensure sustainable wildlife management and ecosystem services for vulnerable communities, One Health has been proposed as an integrated way for a holistic solution. Investment in policy and funding support for alternative development models is essential to contributing to sustainable development goals (SDGs) and other global frameworks. This requires joint actions to address health, wildlife, and livelihood issues and promote sustainable supply chains while discouraging illegal trade in wildlife products. Therefore, the review highlights practical actions demonstrating how One Health fosters and enables transformative change and builds sustainable landscapes and livelihoods (Fig. 3).

## 8 Sustainable growth in green recovery

A strategy and action plan should be developed for green innovations and equitable access, encompassing economic and social services, natural resources, markets, finance, education, and digital technologies. It should also include resource mobilization for sustainable agricultural and food systems, mainly lPLCs in the Asia-Pacific region.

**Fig. 4:**
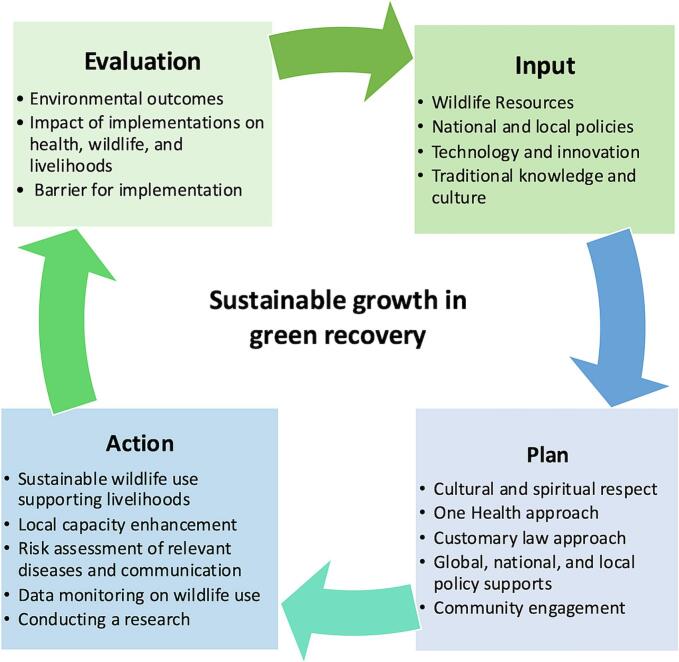
Sustainable growth in green recovery on Health, Wildlife, and Livelihoods. (For interpretation of the references to colour in this figure legend, the reader is referred to the web version of this article.)

Sustainable growth in green recovery is needed to support health, wildlife, and livelihoods. This initiative can ensure access and continue based on the measures outlined in Fig. 4. The activities mentioned earlier on green recovery, and those illustrated in Fig. 1 have been recategorized according to the steps shown in Fig. 4. The progress and effectiveness of the country's campaigns can be evaluated through surveys, focus groups, and other research studies [93].

## 9 Conclusions

Sustainable wildlife management models in the Asia-Pacific region must rely on the active involvement and stewardship of IPLCs. When successfully applied, such models can reduce poverty, improve food security, and prevent the spread of diseases between humans and wildlife. Effective local communication is essential for educating and raising awareness about protecting wildlife and ecosystems and risks for zoonotic disease transmission. Doing so can maintain a balanced ecosystem and safeguard against biodiversity loss. Law enforcement and policies should leverage customary norms and rules to increase their effectiveness and acceptance by IPLCs. Implementing sustainable business practices, such as greening the supply chain and creating shared value, is also necessary. Understanding stakeholder and value chain analyses will help ensure local communities benefit from these efforts.

## Ethics approval and consent to participate

No study participants participated.

## Consent for publication

All authors participated in the development of the manuscript and consented to publication.

## Funding

Food and Agriculture Organization of the United Nations (FAO) supported the development of a discussion paper on the related theme. The review draws several pieces of information from it.

## CRediT authorship contribution statement

**Nareerat Sangkachai:** Writing – review & editing, Writing – original draft, Methodology, Investigation, Data curation, Conceptualization. **Anuwat Wiratsudakul:** Writing – review & editing, Writing – original draft, Methodology, Investigation, Conceptualization. **Delia G. Randolph:** Writing – review & editing, Writing – original draft, Validation. **Maxine Whittaker:** Writing – review & editing, Writing – original draft, Validation. **Acty George:** Writing – review & editing, Writing – original draft, Validation. **Martin R. Nielsen:** Writing – review & editing, Writing – original draft, Validation. **Nicholas Hogarth:** Writing – review & editing, Writing – original draft, Validation. **Dirk U. Pfeiffer:** Writing – review & editing, Writing – original draft, Validation. **Carsten Smith-Hall:** Writing – review & editing, Writing – original draft, Validation. **P.O. Nameer:** Writing – review & editing, Writing – original draft, Validation. **Latiffah Hassan:** Writing – review & editing, Writing – original draft, Validation. **Gautam Talukdar:** Writing – review & editing, Writing – original draft, Validation. **Tien Ming Lee:** Writing – review & editing, Writing – original draft, Validation. **Vinod B. Mathur:** Writing – review & editing, Writing – original draft, Validation. **Innocent B. Rwego:** Writing – review & editing, Writing – original draft, Validation. **James Compton:** Writing – review & editing, Writing – original draft, Validation. **Manon Mispiratceguy:** Writing – review & editing, Writing – original draft, Validation. **Jianbin Shi:** Writing – review & editing, Writing – original draft, Validation. **Amanda E. Fine:** Writing – review & editing, Writing – original draft, Validation. **Illias Animon:** Writing – review & editing, Writing – original draft, Validation, Supervision, Conceptualization. **Kristina Rodina de Carvalho:** Writing – review & editing, Writing – original draft, Validation. **Andrew Taber:** Writing – review & editing, Writing – original draft, Validation. **Scott Newman:** Writing – review & editing, Writing – original draft, Validation, Conceptualization. **Metawee Thongdee:** Writing – review & editing, Writing – original draft, Validation, Methodology, Data curation. **Ladawan Sariya:** Writing – review & editing, Writing – original draft, Validation, Methodology, Data curation. **Siriporn Tangsudjai:** Writing – review & editing, Writing – original draft, Validation, Methodology, Data curation. **Waruja Korkijthamkul:** Writing – review & editing, Writing – original draft, Validation, Data curation. **Walasinee Sakcamduang:** Writing – review & editing, Writing – original draft, Validation, Methodology, Conceptualization. **Sarin Suwanpakdee:** Writing – review & editing, Writing – original draft, Visualization, Project administration, Methodology, Investigation, Data curation, Conceptualization.

## Declaration of competing interest

The authors declare the following financial interests/personal relationships which may be considered as potential competing interests:

Sarin Suwanpakdee reports financial support was provided by Food and Agriculture Organization of the United Nations. If there are other authors, they declare that they have no known competing financial interests or personal relationships that could have appeared to influence the work reported in this paper.

## Data Availability

No original data are presented.

## Appendix A Supplementary data

Supplementary material 1: Demographics of Indigenous People in the Asia-Pacific Region. Supplementary material 1

Supplementary material 2: List of Wildlife Species Harvested for Nutritional Security, Traditional Medicine, and Trade by Indigenous People and Local Communities in the Asia-Pacific Region. Supplementary material 2
