# Supplementary material for: Advancing green recovery: Integrating one health in sustainable wildlife management in the Asia-Pacific Indigenous People and Local Communities
Source: One Health. 2025 Jan 9;20:100969. doi: 10.1016/j.onehlt.2025.100969 (PMC11782897; doi:10.1016/j.onehlt.2025.100969)
Supplement: Supplementary file 1 — Supplementary material 1: Demographics of Indigenous People in the Asia-Pacific Region. [file mmc1.docx]

**Table S1.** Demographics of Indigenous People in the Asia-Pacific Region

| Country | No. of IPLCs | Population | Percentage of total population | References |
| --- | --- | --- | --- | --- |
| Bangladesh | 54 | 1,650,159 | 1 | (1) |
| Cambodia | 24 | 170,000 - 400,000 | 1.1 - 3 | (1) |
| China | 55 | 125,332,335 | 8.89 | (1) |
| India | 705 | 104,000,000 | 8.6 | (1) |
| Indonesia | 2,512 | 50,000,000 - 70,000,000 | 20 - 28 | (1) |
| Laos | 160 | 3,000,000 | 42 | (1, 2) |
| Malaysia | 51 | 3,564,000 | 11 | (1) |
| Myanmar | 135 | 17,000,000 | 33 | (3) |
| Nepal | 63 | 10,872,000 | 36 | (1) |
| Philippines | N/A | 11,300,000 | 10-20 | (1, 4) |
| Thailand | N/A | 5,000,000 | 7.2 | (1) |
| Vietnam | 54 | 13,400,000 | 14 | (5) |
| New Zealand | 1 | 775,500 | 16.5 | (6) |
| Australia | 270 | 812,000 | 3.3 | (7) |
| Bhutan | 4 | 756,000 | 98 | (8) |
| Brunei Darussalam |  | 460,000 | 17 | (9) |
| Iran |  | 31,200,000 | 39 | (10) |
| Japan | 2 | 1,424,000 | 1.13 | (11) |
| Mongolia |  | 301,000 | 10.2 | (12) |
| Afghanistan |  | 30,000,000 |  | (13) |

**References**

1. Mamo D. The Indigenous World 2023. The International Work Group for Indigenous Affairs (IWGIA); 2023.

2. Group MR. Laos 2018 [updated July 2018. Available from: <https://minorityrights.org/country/laos/>.

3. Group MR. Myanmar 2020 [updated November 2020. Available from: <https://minorityrights.org/country/myanmar/>.

4. Group MR. Philippines 2020 [updated May 2020; cited 2024 05]. Available from: <https://minorityrights.org/country/philippines/>.

5. Group MR. Vietnam 2018 [updated March 2018; cited 2024 05]. Available from: <https://minorityrights.org/country/vietnam/>.

6. NZ S. 2018 Census totals by topic 2018 [cited 2024 05]. Available from: <https://www.stats.govt.nz/information-releases/2018-census-totals-by-topic-national-highlights-updated/>.

7. Statistics ABo. Population: Census 2021 [cited 2024 05]. Available from: <https://www.abs.gov.au/statistics/people/population/population-census/latest-release#cite-window1>.

8. Group MR. Bhutan 2020 [cited 2024 05]. Available from: <https://minorityrights.org/country/bhutan/>.

9. Group MR. Brunei Darussalam 2020 [updated May 2020; cited 2024 05]. Available from: <https://minorityrights.org/country/brunei-darussalam/>.

10. Group MR. World Directory of Minorities and Indigenous Peoples - Iran 2017 [updated December 2017; cited 2024 05]. Available from: <https://www.refworld.org/docid/4954ce53c.html>.

11. Group MR. Japan 2019 [updated June 2019; cited 2024 05]. Available from: <https://minorityrights.org/country/japan/>.

12. Group MR. Mongolia 2020 [updated May 2020; cited 2024 05]. Available from: <https://minorityrights.org/country/mongolia/>.

13. Group MR. Afghanistan 2021 [updated December 2021; cited 2024 05]. Available from: <https://minorityrights.org/country/afghanistan/>.
