# Supplementary material for: Advancing green recovery: Integrating one health in sustainable wildlife management in the Asia-Pacific Indigenous People and Local Communities
Source: One Health. 2025 Jan 9;20:100969. doi: 10.1016/j.onehlt.2025.100969 (PMC11782897; doi:10.1016/j.onehlt.2025.100969)
Supplement: Supplementary file 2 — Supplementary material 2: List of Wildlife Species Harvested for Nutritional Security, Traditional Medicine, and Trade by Indigenous People and Local Communities in the Asia-Pacific Region. [file mmc2.docx]

**Table S2** List of Wildlife Species Harvested for Nutritional Security, Traditional Medicine, and Trade by Indigenous People and Local Communities in the Asia-Pacific Region

| **Country** | **Wildlife species used by IPLCs** | **Reference** |
| --- | --- | --- |
| Australia | Kangaroo, Saltwater Crocodile, Magpie Goose, Asian Swamp Buffalo, Common Wallaroo, Emu, Dugong, Sea Turtle, Australian bustards, Arafura File Snake, Snapping Turtle, Northern Snake-necked Turtle, and Tarantula spider | (1-3) |
| Bangladesh | Large Indian Civet, Rhesus Macaque, Asian Elephant, Asian Black Bear, Wild Boar, Barking Deer, Sambar Deer, Golden Jackal, Indian Palm Squirrel, House Shrew, Indian Mongoose, Gaur, Junglefowl, Cobras, Indian Softshell Turtle, Indian River Turtles, Roofed Turtles, Indian Flapshell Turtle, Eurasian Collared Dove, Indian Pond Heron, White-breasted Waterhen, Mynas, Sparrow, Little Cormorant, Black-necked Hare, Jungle Cat, Rats, and Bandicoots | (4, 5) |
| Bhutan | Asian Black Bear, Musk Deer, Water Buffalo, Black Vulture, Indian One-horned Rhinoceros, Himalayan Serow, Red Deer, Red Fox, Wild Boar, Common Wolf, Big-eyed Rat Snake, Chinese Mountain Salamander, Asiatic Toad, Agamid Lizard, Tibetan Antelope, Common Sparrow, Hill Pigeon, Pallas's Fish Eagle, Woolly Hare, Wild Yak, Black-billed Magpie, Tiger, Chinese Pangolin, Indian Pangolin, Burmese Python, and Asian Elephant | (6) |
| Cambodia | Water Monitor, Asian Porcupine, Wild Boar, Barking Deer, Slow Loris, Mouse Deer, Turtle, Snake, Squirrel, Civet, Sunda Pangolin, Binturong, Bat, Burmese Hare, Sambar Deer, Northern Tree Shrew, Small Asian Mongoose, Long-tailed Macaque, Junglefowl, Monkey, Fishing Cat, Leopard, Serow, Gaur, Leopard Cat, Otter, Tortoise, Pig-tailed Macaque, Siamese Crocodile, Land Lizard, Reticulated python, Black Bear, Asian Wild Dog, Tiger, Pileated Gibbon, Banteng, Clouded Leopard, Asian Eleplant, Javan Rhinocerous, Silvered Langur, and Sun Bear | (7) |
| China | Silver Pheasant, Red Junglefowl, Partridge, Mountain Imperial Pigeon, Great Barbet, Emerald Dove, Wild Boar, Barking Deer, Flying Squirrel, Mouse Deer, Bamboo Rat, Bat, Palm Civet, Red Panda, Griffon Vulture, Common Pheasant, Water Monitor, Mongolian Gazelle, Tibetan Gazelle, Chiru, Sumatran Serow, Goitered Gazelle, Himalayan Goral, Argali Sheep, Red Deer, Thorold's Deer, Golden Musk Deer, Yak, Kiang, Gray Wolf, Eurasian Lynx, Pallas's Cat, Snow Leopard, Tiger, Himalayan Black Bear, Golden Snub-nosed Monkey, Common Crane, Common Pheasant, Eagles, Snakes, Rhino,  Geckos, Crocodiles, Badgers, Giant salamanders, Hedgehog, Porcupines, and Rabbit | (8-10) |
| India | Himalayan Black Bear, Tiger, Jackal, Red Fox, Wild Dog, Wild Boar, Barking Deer, Himalayan Musk Deer, Spotted Deer, Himalayan Tahr, Rhesus Macaque, Red-giant Flying Squirrel, Porcupine, Indian Hare, Mongooses, King Cobra, Birds, Freshwater Fishes, Sambar Deer, Nilgiri Tahr, Black-footed Gray Langur, Tufted Gray Langur, Nilgiri Langur, Lion-tailed Macaque, Smooth-coated Otter, Indian Mouse deer/chevrotain, Indian Pangolin, Indian-crested Porcupine, Asian Palm Civet, Brown Palm Civet, Small Indian Civet, Malabar Giant Squirrel, Large Brown Flying Squirrel, Travancore Flying Squirrel, Fruit Bats, False Bandicoot, Rat, Bare-bellied Hedgehog, Great Hornbill, Pied hornbill, Malabar Grey Hornbill, Indian Grey Hornbill, Indian Peafowl, Grey Junglefowl, Grey Francolin, Owl, Egret/heron sp., Emerald Dove, Spotted Dove, Pompadour Pigeon, Rock Pigeon, Parakeet, Crow and Bulbul, Indian rock python, Boa, Monitor lizard, Indian flapshell turtle, Travancore tortoise, Indian black turtle, Cochin forest cane turtle, Southern flying lizard, Malabar gliding frog, and Andaman wild pig | (10, 11) |
| Indonesia | Wild Boar, Rusa Deer, Spiny Bandicoot, Clara’s Bandicoot, White-striped Wallaby, Grizzled Tree Kangaroo, Greater Flying Fox, Spotted Cuscus, Ground Cuscus, North-east Cuscus, Peach-throated monitor, Northern Cassowary, and Snakes | (12, 13) |
| Iran | Ibex, Wild sheep, Goitered Gazelle, Jebeer Gazelle, Persian Wild Ass, Wild Boar, Red Deer, Roe Deer, Brown Bear, Hare, Red Fox, Rock Marten, Otter, Chukar, Pheasant, Coot, Mallard, Teal, Pochard, Shoveler, and Pintail | (14) |
| Laos | Birds, Barking Deer, Frogs, Squirrels, Rats, Junglefowl, and Wild Boar | (15) |
| Malaysia | Sunda Pangolin, White-thighed Leaf Monkey, Dusky Leaf Monkey, Pig-tailed Macaque, Long-tailed Macaque, White-handed Gibbon, Wild Dog, Sun Bear, Yellow-throated Marten, Smooth-coated Otter, Small-clawed Otter, Malay Civet, Large Indian Civet, Common Palm Civet, Binturong, Crab-eating Mongoose, Tiger, Leopard, Golden Cat, Leopard Cat, Asian Elephant, Asian Tapir, Wild Boar, Sambar Deer, Barking Deer, and Grey-bellied Squirrel | (16) |
| Mongolia | Musk Deer, Red Deer, Brown Bear, Wild Boar, Roe Deer, Marmot, Badger, Mongolian Gazelle, Saiga Antelope, Argali, Siberia Marmot, Ibex, Mongolian wild ass, Moose, and Reindeer | (17-19) |
| Myanmar | Malayan Porcupine, Irrawaddy Dolphin, Pangolin, Macaque, Gibbons, Asiatic Black Bear, Sun Bear, Binturong, Small Indian Civet, Otter, Leopard, Clouded Leopard, Tiger, Asian Elephant, Wild Boar, Barking Deer, Chinese Serow, Red Goral, Red Panda, Takin, Pheasant, Yellow-throated Marten, Monitor Lizard, Snake, Deer, Porcupine, Flying fox, Hornbill, Crocodile, Bamboo Rat, Common Palm Civet,Gaur, Jungle Cat, Spotted Linsang, Turtle, Squirrel, Junglefowl, and Slow Loris | (20-22) |
| Nepal | Babbler, Sparrow, Hornbill, Raptors, Kite, Pigeon,  Partridges, Kalij pheasant, Rhinoceros, Elephant, Tiger, Bear, Leopard, Barking deer, Otter, Porcupine, Jackal, Monkey, Mongoose, Pangolin,  and Four-horned Antelope | (23) |
| New Zealand | Pacific Oyster, Chilean Flat Oyster, Sea Cucumbers, Eels, Slipper Lobster, and CrayFish | (24) |
| Papua New Guinea | Birds, Echidna, Quoll, Bandicoot, Cuscus, Striped Possum, Tree-kangaroo, Wallaby, Fruit bat, Wild Boar, Cassowary, Long-nosed Mellomys, Moss Forest Rat, Speckled Dasyure, and Ringtail Possum | (25, 26) |
| Philippines | Philippine Brown Deer, Common Palm Civet, Reticulated Python, Monitor Lizard, Philippine Cobra, King Cobra, Birds, Wild Boar, Philippine Warty Pig, Junglefowl, Dove, Hornbill, Flying fox, Fruit Bat, Small Flying Lemurs, Tarsier, and Long-tailed Macaques | (27) |
| Solomon Islands | Rousette Bats, Leaf-nosed Bats, Hawksbill Sea Turtle, Solomon Flying fox, and Wild Boar | (28, 29) |
| Sri Lanka | Water Tortoise, Mouse Deer, Cobra, and Oysters | (30) |
| Thailand | Bats, Monitor Lizard, Snake, Deer, Porcupine, Asiatic Black Bear, Flying fox, and Hornbill | (31) |
| Timor Leste | Civet, Deer, Jungle fowl, and Monkey | (32) |
| Vietnam | Wild Boar, Wild Goat, Deer, Snake, Civet, Serow, Barking Deer, Frogs, Turtles, Porcupine, Monitor Lizard, Rabbits, Pangolin, Wild Cat, Gecko, Monkey, Tiger, Ostrich, Crocodile, Reticulated Python, Junglefowl, Dove, Hornbill, Flying fox, Deer, Mouse, and Fish | (33) |
| Vanuatu | Wild Boar, Fruit Bats, Turtles, and Pelagic Birds | (34) |
| Other Pacific Islands (Fiji, Kiribati, Marshall Island, Micronesia,) | Melon-headed Whale, Freshwater Eels, Sea Cucumber, Land Crab, Giant Clams, Pacific Yellowtail Emperor, Parrotfish, Spotcheek Emperor, and Orangespine Unicornfish | (35-37) |

*Note: Please note that some examples above are highly site/community-specific*

**References**

1. Gorman JT, Whitehead PJ, Griffiths AD, Petheram L. Production from marginal lands: indigenous commercial use of wild animals in northern Australia. The International Journal of Sustainable Development & World Ecology. 2008;15(3):240-50.

2. Fordham A, Fogarty W, Fordham D. The viability of wildlife enterprises in remote indigenous communities of Australia: a case study: Canberra, ACT: Centre for Aboriginal Economic Policy Research (CAEPR), The …; 2010.

3. Wilson GR, Edwards MJ, Smits JK. Support for Indigenous wildlife management in Australia to enable sustainable use. Wildlife Research. 2010;37(3):255-63.

4. Chowdhury MSH, Izumiyama S, Nazia N, Muhammed N, Koike M. Dietetic use of wild animals and traditional cultural beliefs in the Mro community of Bangladesh: an insight into biodiversity conservation. Biodiversity. 2014;15(1):23-38.

5. Barkat AI, Liza FT, Akter S, Shome AR, Rabbe MF. Wildlife hunting practices of the Santal and Oraon communities in Rajshahi, Bangladesh. Journal of Threatened Taxa. 2021;13(11):19484-91.

6. Yeshi K, Morisco P, Wangchuk P. Animal-derived natural products of Sowa Rigpa medicine: Their pharmacopoeial description, current utilization and zoological identification. Journal of ethnopharmacology. 2017;207:192-202.

7. Coad L, Lim S, Nuon L. Wildlife and livelihoods in the Cardamom Mountains, Cambodia. Frontiers in Ecology and Evolution. 2019;7:296.

8. Commerçon FA, Zhang M, Solomon JN. Social norms shape wild bird hunting: A case study from southwest China. Global Ecology and Conservation. 2021;32:e01882.

9. Yi-Ming L, Zenxiang G, Xinhai L, Sung W, Niemelä J. Illegal wildlife trade in the Himalayan region of China. Biodiversity & Conservation. 2000;9:901-18.

10. Mainka SA, Mills JA. Wildlife and traditional Chinese medicine: supply and demand for wildlife species. Journal of zoo and wildlife medicine. 1995:193-200.

11. Dhakal P, Chettri B, Lepcha S, Acharya BK. Rich yet undocumented ethnozoological practices of socio-culturally diverse indigenous communities of Sikkim Himalaya, India. Journal of ethnopharmacology. 2020;249:112386.

12. Pangau-Adam M, Noske R. Wildlife hunting and bird trade in northern Papua (Irian Jaya), Indonesia. Ethno. 2010:73.

13. Pangau-Adam M, Noske R, Muehlenberg M. Wildmeat or bushmeat? Subsistence hunting and commercial harvesting in Papua (West New Guinea), Indonesia. Human Ecology. 2012;40:611-21.

14. De Vos A, editor The present and potential significance of wildlife resources to the economy of Iran. Proceedings of the an International Meeting on Ecological Guidelines for the Use of Natural Resources in the Middle East and South West Asia; 1975.

15. Pathumphone S, Sanitchon J, Polthanee A, Promkhambut A. Food diversity of three ethnic groups: A case study from Xieng Khuang province, Northern Lao PDR. Asia Pac J Sci Technol. 2016;21:12-25.

16. Bartholomew CV, Zainir MI, Nor Zalipah M, Husin MH, Abdullah MT. Wildlife hunting practices by the indigenous people of Terengganu, Peninsular Malaysia. Resource use and sustainability of Orang Asli: Indigenous communities in Peninsular Malaysia. 2021:137-53.

17. Pratt D, Macmillan DC, Gordon IJ. Local community attitudes to wildlife utilisation in the changing economic and social context of Mongolia. Biodiversity & Conservation. 2004;13:591-613.

18. Zahler P, Lhagvasuren B, Reading RP, Wingard JR, Amgalanbaatar S, Gombobaatar S, et al. Illegal and unsustainable wildlife hunting and trade in Mongolia. Mongolian Journal of Biological Sciences. 2004;2(2):23-31.

19. Olson KA, Fuller TK. Wildlife hunting in eastern Mongolia: Economic and demographic factors influencing hunting behavior of herding households. Mongolian Journal of Biological Sciences. 2017;15(1-2):37-46.

20. Rao M, Zaw T, Htun S, Myint T. Hunting for a living: Wildlife trade, rural livelihoods and declining wildlife in the Hkakaborazi National Park, North Myanmar. Environmental Management. 2011;48(1):158-67.

21. Nijman V, Shepherd CR. Ethnozoological assessment of animals used by Mon traditional medicine vendors at Kyaiktiyo, Myanmar. Journal of ethnopharmacology. 2017;206:101-6.

22. McEvoy JF, Connette G, Huang Q, Soe P, Pyone KHH, Valitutto M, et al. Two sides of the same coin–Wildmeat consumption and illegal wildlife trade at the crossroads of Asia. Biological Conservation. 2019;238:108197.

23. Chalise MK. SUSTAINABLE USAGE OF ANIMALS BY THE RURAL PEOPLE IN REFERENCE TO NEPAL. 2010.

24. Alfaro AC, Jeffs AG, King N. Enabling and driving aquaculture growth in New Zealand through innovation. Taylor & Francis; 2014. p. 311-3.

25. Cuthbert R. Sustainability of hunting, population densities, intrinsic rates of increase and conservation of Papua New Guinean mammals: a quantitative review. Biological Conservation. 2010;143(8):1850-9.

26. Sillitoe P, editor Always been farmer-foragers? Hunting and gathering in the Papua New Guinea Highlands. Anthropological Forum; 2002: Taylor & Francis.

27. Tanalgo KC. Wildlife hunting by indigenous people in a Philippine protected area: a perspective from Mt. Apo National Park, Mindanao Island. Journal of Threatened Taxa. 2017;9(6):10307-13.

28. Lavery T, Pikacha P, Fisher D. Solomon Islands forest life: information on biology and management of forest resources. 2016.

29. Vuto S, Hamilton R, Brown C, Waldie P, Pita J, Peterson N, et al. A report on turtle harvest and trade in Solomon Islands. Solomon Island: The Nature Conservancy. 2019;34.

30. Kankanamalage T, Dharmadasa R, Abeysinghe D, Wijesekara R. A survey on medicinal materials used in traditional systems of medicine in Sri Lanka. Journal of ethnopharmacology. 2014;155(1):679-91.

31. Somnasang P, Moreno G, Chusil K. Indigenous knowledge of wild food hunting and gathering in north-east Thailand. Food and Nutrition Bulletin. 1998;19(4):359-65.

32. Wong JT, Bagnol B, Grieve H, da Costa Jong JB, Li M, Alders RG. Factors influencing animal-source food consumption in Timor-Leste. Food Security. 2018;10:741-62.

33. Sandalj M, Treydte AC, Ziegler S. Is wild meat luxury? Quantifying wild meat demand and availability in Hue, Vietnam. Biological Conservation. 2016;194:105-12.

34. Valentin F, Buckley HR, Herrscher E, Kinaston R, Bedford S, Spriggs M, et al. Lapita subsistence strategies and food consumption patterns in the community of Teouma (Efate, Vanuatu). Journal of Archaeological Science. 2010;37(8):1820-9.

35. Robards MD, Reeves RR. The global extent and character of marine mammal consumption by humans: 1970–2009. Biological Conservation. 2011;144(12):2770-86.

36. Taylor B, Choat J. Comparative demography of commercially important parrotfish species from Micronesia. Journal of fish biology. 2014;84(2):383-402.

37. Pardee C, Taylor BM, Felise S, Ochavillo D, Cuetos-Bueno J. Growth and maturation of three commercially important coral reef species from American Samoa. Fisheries science. 2020;86(6):985-93.
